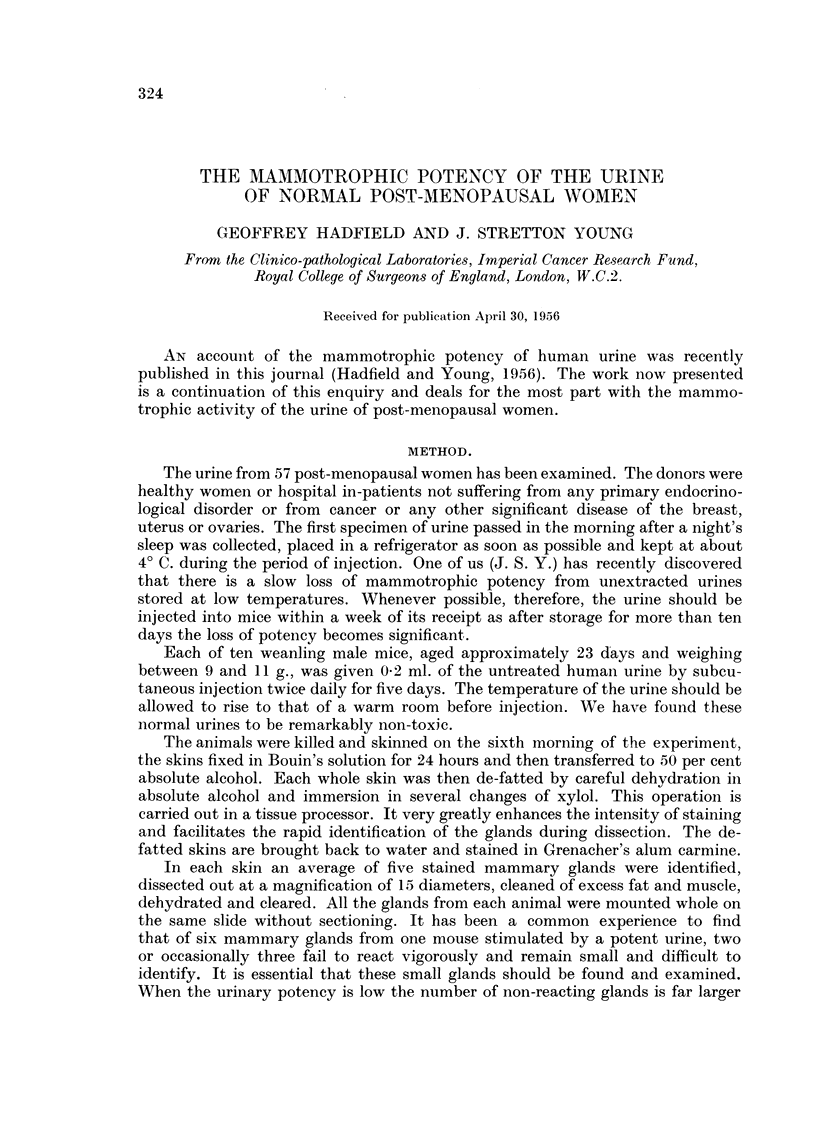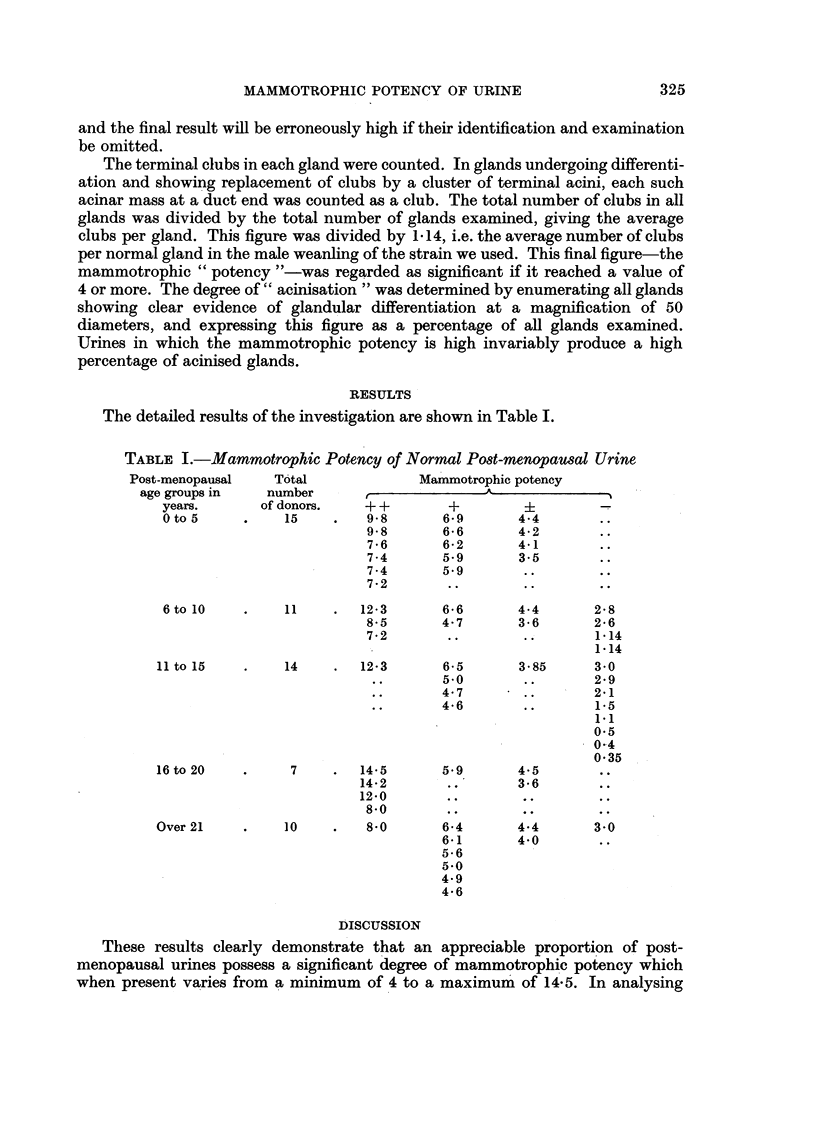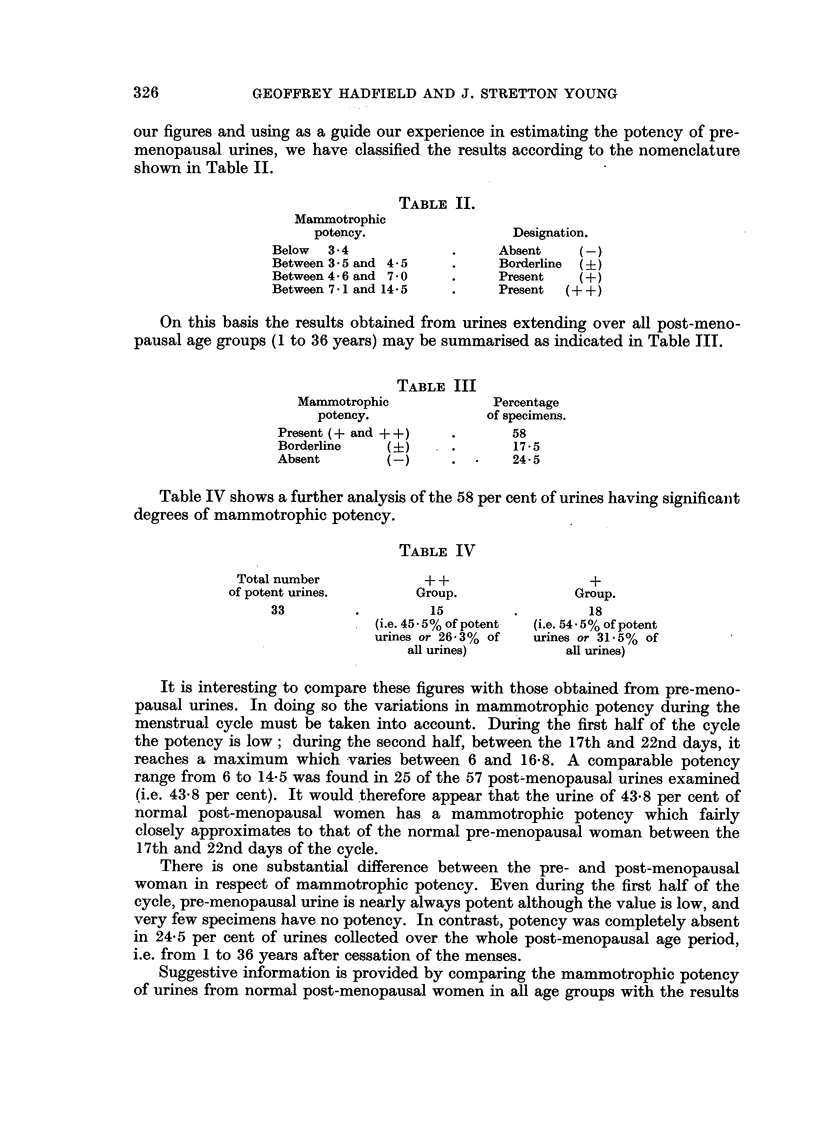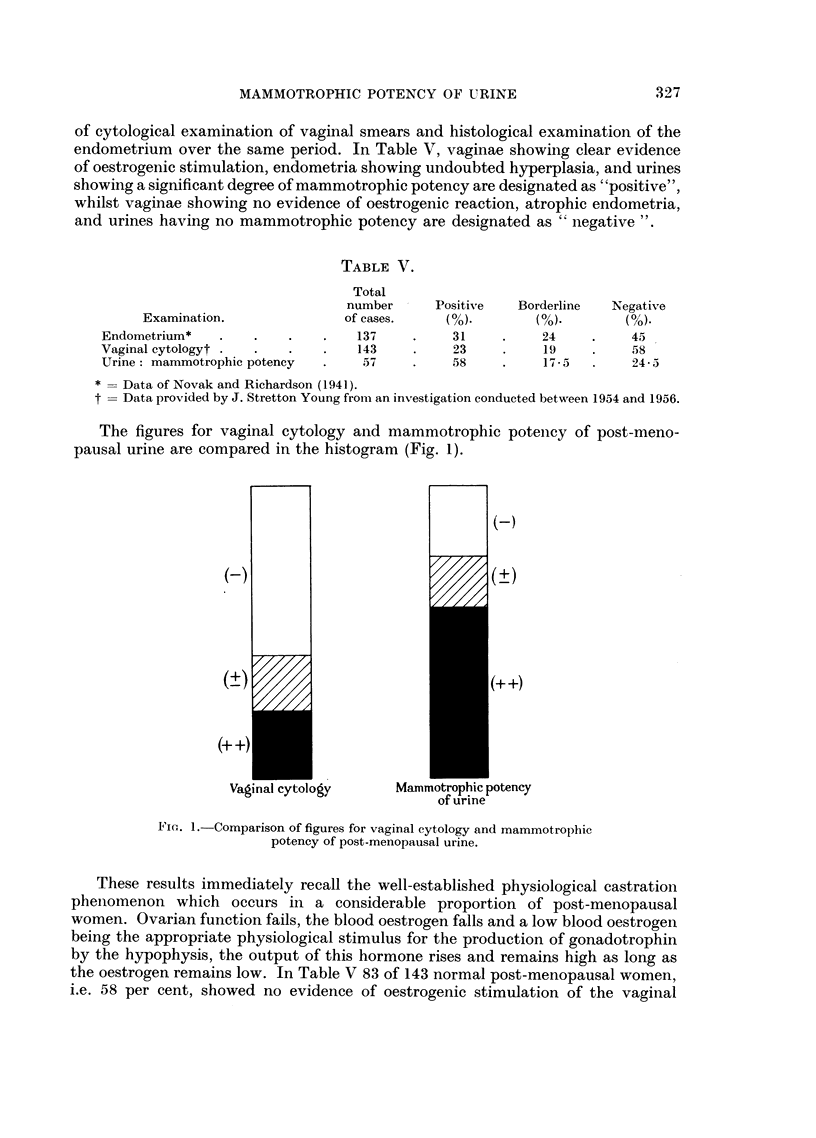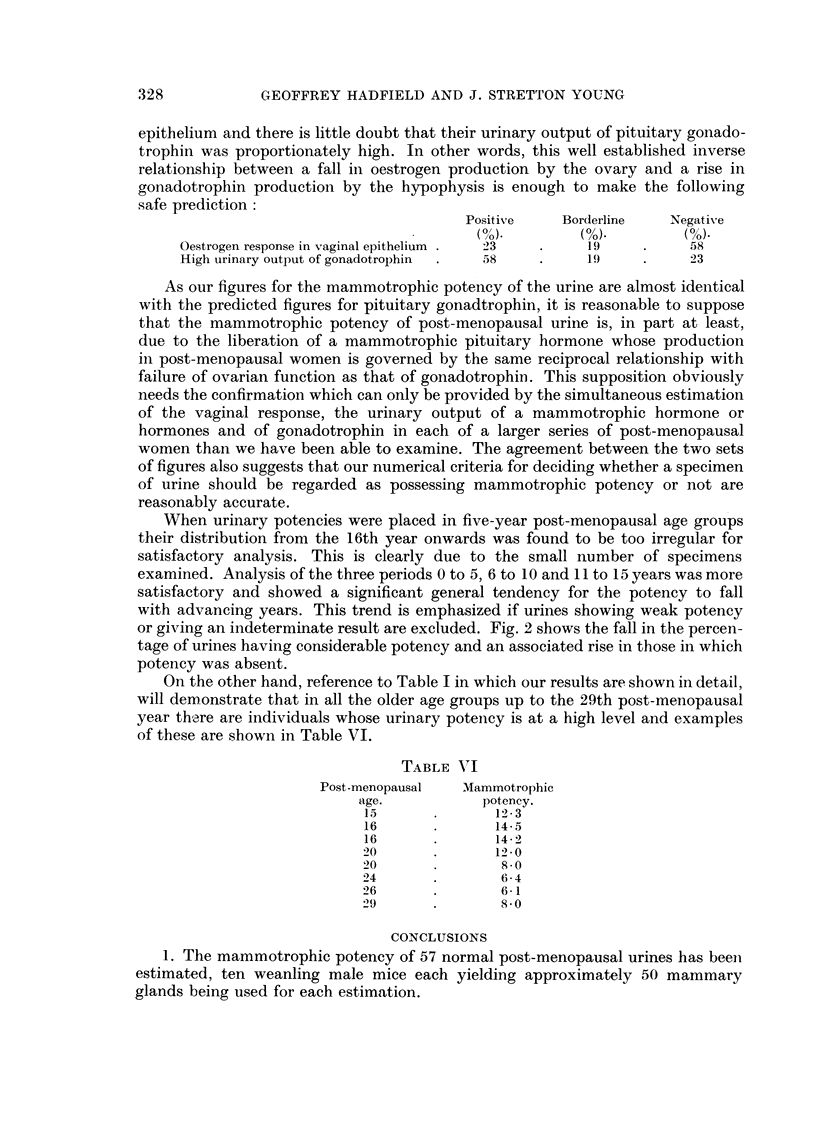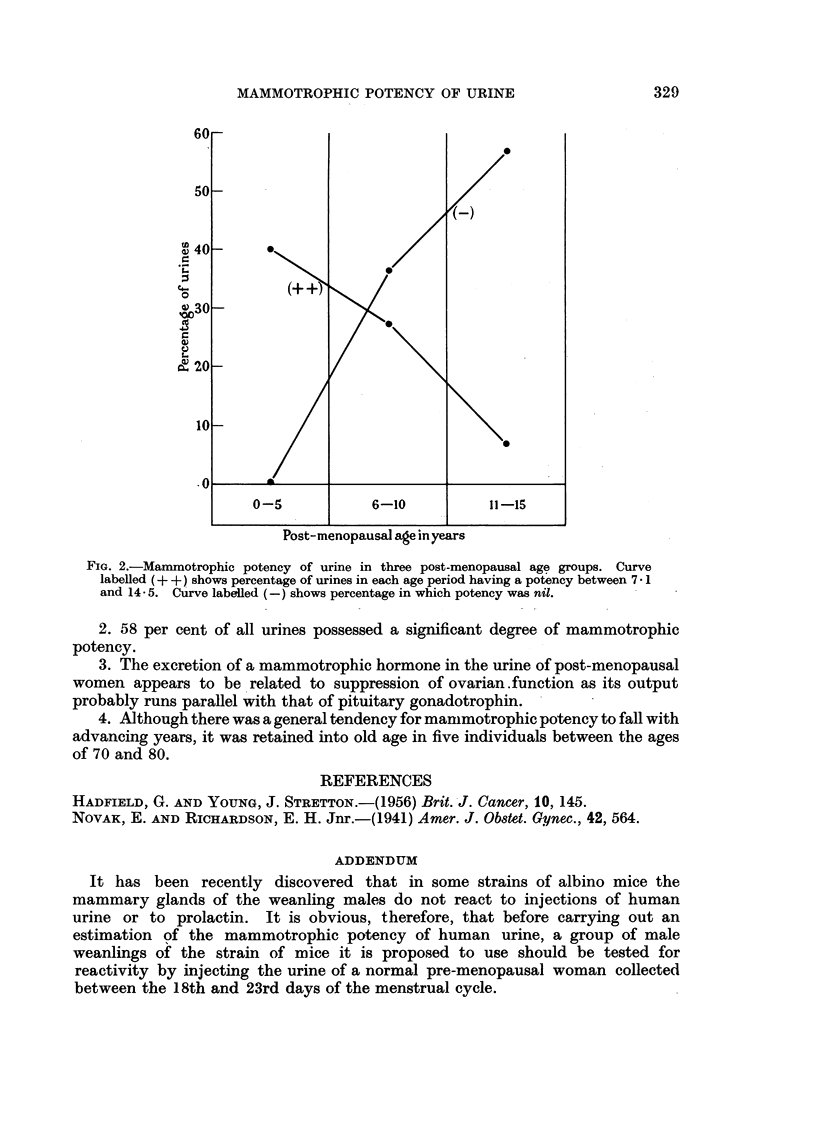# The Mammotrophic Potency of the Urine of Normal Post-Menopausal Women

**DOI:** 10.1038/bjc.1956.39

**Published:** 1956-06

**Authors:** Geoffrey Hadfield, J. Stretton Young


### THE MAMMOTROPHIC POTENCY OF THE URINE OF NORMAL POST-MENOPAUSAL WOMEN

GEOFFREY HADFIELD AND J. STRETTON YOUNG

*From the Clinico-pathological Laboratories, Imperial Cancer Research Fund, Royal College of Surgeons of England, London, W.C.2.*

Received for publication April 30, 1956

AN account of the mammotrophic potency of human urine was recently published in this journal (Hadfield and Young, 1956). The work now presented is a continuation of this enquiry and deals for the most part with the mammotrophic activity of the urine of post-menopausal women.

### METHOD.

The urine from 57 post-menopausal women has been examined. The donors were healthy women or hospital in-patients not suffering from any primary endocrinological disorder or from cancer or any other significant disease of the breast, uterus or ovaries. The first specimen of urine passed in the morning after a night's sleep was collected, placed in a refrigerator as soon as possible and kept at about 4° C. during the period of injection. One of us (J. S. Y.) has recently discovered that there is a slow loss of mammotrophic potency from unextracted urines stored at low temperatures. Whenever possible, therefore, the urine should be injected into mice within a week of its receipt as after storage for more than ten days the loss of potency becomes significant.

Each of ten weanling male mice, aged approximately 23 days and weighing between 9 and 11 g., was given 0·2 ml. of the untreated human urine by subcutaneous injection twice daily for five days. The temperature of the urine should be allowed to rise to that of a warm room before injection. We have found these normal urines to be remarkably non-toxic.

The animals were killed and skinned on the sixth morning of the experiment, the skins fixed in Bouin's solution for 24 hours and then transferred to 50 per cent absolute alcohol. Each whole skin was then de-fatted by careful dehydration in absolute alcohol and immersion in several changes of xylol. This operation is carried out in a tissue processor. It very greatly enhances the intensity of staining and facilitates the rapid identification of the glands during dissection. The de-fatted skins are brought back to water and stained in Grenacher's alum carmine.

In each skin an average of five stained mammary glands were identified, dissected out at a magnification of 15 diameters, cleaned of excess fat and muscle, dehydrated and cleared. All the glands from each animal were mounted whole on the same slide without sectioning. It has been a common experience to find that of six mammary glands from one mouse stimulated by a potent urine, two or occasionally three fail to react vigorously and remain small and difficult to identify. It is essential that these small glands should be found and examined. When the urinary potency is low the number of non-reacting glands is far larger and the final result will be erroneously high if their identification and examination be omitted.

The terminal clubs in each gland were counted. In glands undergoing differentiation and showing replacement of clubs by a cluster of terminal acini, each such acinar mass at a duct end was counted as a club. The total number of clubs in all glands was divided by the total number of glands examined, giving the average clubs per gland. This figure was divided by 1·14, i.e. the average number of clubs per normal gland in the male weanling of the strain we used. This final figure—the mammotrophic " potency "—was regarded as significant if it reached a value of 4 or more. The degree of " acinisation " was determined by enumerating all glands showing clear evidence of glandular differentiation at a magnification of 50 diameters, and expressing this figure as a percentage of all glands examined. Urines in which the mammotrophic potency is high invariably produce a high percentage of acinised glands.

### RESULTS

The detailed results of the investigation are shown in Table I.

TABLE I.—*Mammotrophic Potency of Normal Post-menopausal Urine*

| Post-menopausal age groups in years. | Total number of donors. | Mammotrophic potency | | | |
|---|---|---|---|---|---|
| | | ++ | + | ± | − |
| 0 to 5 | 15 | 9·8 | 6·9 | 4·4 | .. |
| | | 9·8 | 6·6 | 4·2 | .. |
| | | 7·6 | 6·2 | 4·1 | .. |
| | | 7·4 | 5·9 | 3·5 | .. |
| | | 7·4 | 5·9 | .. | .. |
| | | 7·2 | .. | .. | .. |
| 6 to 10 | 11 | 12·3 | 6·6 | 4·4 | 2·8 |
| | | 8·5 | 4·7 | 3·6 | 2·6 |
| | | 7·2 | .. | .. | 1·14 |
| | | | | | 1·14 |
| 11 to 15 | 14 | 12·3 | 6·5 | 3·85 | 3·0 |
| | | .. | 5·0 | .. | 2·9 |
| | | .. | 4·7 | .. | 2·1 |
| | | .. | 4·6 | .. | 1·5 |
| | | | | | 1·1 |
| | | | | | 0·5 |
| | | | | | 0·4 |
| | | | | | 0·35 |
| 16 to 20 | 7 | 14·5 | 5·9 | 4·5 | .. |
| | | 14·2 | .. | 3·6 | .. |
| | | 12·0 | .. | .. | .. |
| | | 8·0 | .. | .. | .. |
| Over 21 | 10 | 8·0 | 6·4 | 4·4 | 3·0 |
| | | | 6·1 | 4·0 | .. |
| | | | 5·6 | | |
| | | | 5·0 | | |
| | | | 4·9 | | |
| | | | 4·6 | | |

### DISCUSSION

These results clearly demonstrate that an appreciable proportion of post-menopausal urines possess a significant degree of mammotrophic potency which when present varies from a minimum of 4 to a maximum of 14·5. In analysing our figures and using as a guide our experience in estimating the potency of pre-menopausal urines, we have classified the results according to the nomenclature shown in Table II.

TABLE II.

| Mammotrophic potency. | Designation. |
|---|---|
| Below 3·4 | Absent (−) |
| Between 3·5 and 4·5 | Borderline (±) |
| Between 4·6 and 7·0 | Present (+) |
| Between 7·1 and 14·5 | Present (++) |

On this basis the results obtained from urines extending over all post-menopausal age groups (1 to 36 years) may be summarised as indicated in Table III.

TABLE III

| Mammotrophic potency. | Percentage of specimens. |
|---|---|
| Present (+ and ++) | 58 |
| Borderline (±) | 17·5 |
| Absent (−) | 24·5 |

Table IV shows a further analysis of the 58 per cent of urines having significant degrees of mammotrophic potency.

TABLE IV

| Total number of potent urines. | ++ Group. | + Group. |
|---|---|---|
| 33 | 15 (i.e. 45·5% of potent urines *or* 26·3% of all urines) | 18 (i.e. 54·5% of potent urines *or* 31·5% of all urines) |

It is interesting to compare these figures with those obtained from pre-menopausal urines. In doing so the variations in mammotrophic potency during the menstrual cycle must be taken into account. During the first half of the cycle the potency is low ; during the second half, between the 17th and 22nd days, it reaches a maximum which varies between 6 and 16·8. A comparable potency range from 6 to 14·5 was found in 25 of the 57 post-menopausal urines examined (i.e. 43·8 per cent). It would therefore appear that the urine of 43·8 per cent of normal post-menopausal women has a mammotrophic potency which fairly closely approximates to that of the normal pre-menopausal woman between the 17th and 22nd days of the cycle.

There is one substantial difference between the pre- and post-menopausal woman in respect of mammotrophic potency. Even during the first half of the cycle, pre-menopausal urine is nearly always potent although the value is low, and very few specimens have no potency. In contrast, potency was completely absent in 24·5 per cent of urines collected over the whole post-menopausal age period, i.e. from 1 to 36 years after cessation of the menses.

Suggestive information is provided by comparing the mammotrophic potency of urines from normal post-menopausal women in all age groups with the results of cytological examination of vaginal smears and histological examination of the endometrium over the same period. In Table V, vaginae showing clear evidence of oestrogenic stimulation, endometria showing undoubted hyperplasia, and urines showing a significant degree of mammotrophic potency are designated as "positive", whilst vaginae showing no evidence of oestrogenic reaction, atrophic endometria, and urines having no mammotrophic potency are designated as " negative ".

TABLE V.

| Examination. | Total number of cases. | Positive (%). | Borderline (%). | Negative (%). |
|---|---|---|---|---|
| Endometrium* | 137 | 31 | 24 | 45 |
| Vaginal cytology† | 143 | 23 | 19 | 58 |
| Urine : mammotrophic potency | 57 | 58 | 17·5 | 24·5 |

* = Data of Novak and Richardson (1941).
† = Data provided by J. Stretton Young from an investigation conducted between 1954 and 1956.

The figures for vaginal cytology and mammotrophic potency of post-menopausal urine are compared in the histogram (Fig. 1).

FIG. 1.—Comparison of figures for vaginal cytology and mammotrophic potency of post-menopausal urine.

These results immediately recall the well-established physiological castration phenomenon which occurs in a considerable proportion of post-menopausal women. Ovarian function fails, the blood oestrogen falls and a low blood oestrogen being the appropriate physiological stimulus for the production of gonadotrophin by the hypophysis, the output of this hormone rises and remains high as long as the oestrogen remains low. In Table V 83 of 143 normal post-menopausal women, i.e. 58 per cent, showed no evidence of oestrogenic stimulation of the vaginal epithelium and there is little doubt that their urinary output of pituitary gonadotrophin was proportionately high. In other words, this well established inverse relationship between a fall in oestrogen production by the ovary and a rise in gonadotrophin production by the hypophysis is enough to make the following safe prediction :

| | Positive (%). | Borderline (%). | Negative (%). |
|---|---|---|---|
| Oestrogen response in vaginal epithelium | 23 | 19 | 58 |
| High urinary output of gonadotrophin | 58 | 19 | 23 |

As our figures for the mammotrophic potency of the urine are almost identical with the predicted figures for pituitary gonadtrophin, it is reasonable to suppose that the mammotrophic potency of post-menopausal urine is, in part at least, due to the liberation of a mammotrophic pituitary hormone whose production in post-menopausal women is governed by the same reciprocal relationship with failure of ovarian function as that of gonadotrophin. This supposition obviously needs the confirmation which can only be provided by the simultaneous estimation of the vaginal response, the urinary output of a mammotrophic hormone or hormones and of gonadotrophin in each of a larger series of post-menopausal women than we have been able to examine. The agreement between the two sets of figures also suggests that our numerical criteria for deciding whether a specimen of urine should be regarded as possessing mammotrophic potency or not are reasonably accurate.

When urinary potencies were placed in five-year post-menopausal age groups their distribution from the 16th year onwards was found to be too irregular for satisfactory analysis. This is clearly due to the small number of specimens examined. Analysis of the three periods 0 to 5, 6 to 10 and 11 to 15 years was more satisfactory and showed a significant general tendency for the potency to fall with advancing years. This trend is emphasized if urines showing weak potency or giving an indeterminate result are excluded. Fig. 2 shows the fall in the percentage of urines having considerable potency and an associated rise in those in which potency was absent.

On the other hand, reference to Table I in which our results are shown in detail, will demonstrate that in all the older age groups up to the 29th post-menopausal year there are individuals whose urinary potency is at a high level and examples of these are shown in Table VI.

TABLE VI

| Post-menopausal age. | Mammotrophic potency. |
|---|---|
| 15 | 12·3 |
| 16 | 14·5 |
| 16 | 14·2 |
| 20 | 12·0 |
| 20 | 8·0 |
| 24 | 6·4 |
| 26 | 6·1 |
| 29 | 8·0 |

### CONCLUSIONS

1. The mammotrophic potency of 57 normal post-menopausal urines has been estimated, ten weanling male mice each yielding approximately 50 mammary glands being used for each estimation.

FIG. 2.—Mammotrophic potency of urine in three post-menopausal age groups. Curve labelled (+ +) shows percentage of urines in each age period having a potency between 7·1 and 14·5. Curve labelled (—) shows percentage in which potency was *nil*.

2. 58 per cent of all urines possessed a significant degree of mammotrophic potency.

3. The excretion of a mammotrophic hormone in the urine of post-menopausal women appears to be related to suppression of ovarian function as its output probably runs parallel with that of pituitary gonadotrophin.

4. Although there was a general tendency for mammotrophic potency to fall with advancing years, it was retained into old age in five individuals between the ages of 70 and 80.

### REFERENCES

HADFIELD, G. AND YOUNG, J. STRETTON.—(1956) *Brit. J. Cancer*, **10**, 145.
NOVAK, E. AND RICHARDSON, E. H. Jnr.—(1941) *Amer. J. Obstet. Gynec.*, **42**, 564.

### ADDENDUM

It has been recently discovered that in some strains of albino mice the mammary glands of the weanling males do not react to injections of human urine or to prolactin. It is obvious, therefore, that before carrying out an estimation of the mammotrophic potency of human urine, a group of male weanlings of the strain of mice it is proposed to use should be tested for reactivity by injecting the urine of a normal pre-menopausal woman collected between the 18th and 23rd days of the menstrual cycle.